# A Word to M.Ps.

**Published:** 1888-07-14

**Authors:** 


// The Hospital

A Weekly Institutional Journal of

Science, Medicine, Nursing, and Philanthropy.

*For Hospitals, Asylums, Medical Practitioners, Students, Nurses, Families, Parishes, Congregations, and the Charitable Public.*

Vol. IV. No. 94.] [July 14, 1888.

### A Word to M.P.s.

It may be taken for granted that the average M.P. is a man well disposed toward the public in general. He would by preference choose to do his constituency as many good turns as possible. It would not merely "pay" him, from an electioneering standpoint, to do so; but it would give him that pleasure which good deeds generally produce, and would probably secure for him some of that popularity which to ordinary men is not only incense, but a kind of beatitude. The undeveloped Member of Parliament has not as yet quite gone about his work in his constituency in the way to secure the highest measure of personal popularity. He is often a bird of passage, and is much on the wing. His visits, except at election times, are like angel's—"short and far between." There are certain landmarks which ought to be as familiar to him as the churchyard is to the parson or the market-place to the farmer. He should know, with an intimate knowledge, all about the churches and chapels, the reading-rooms and literary institutes, the cricket and football clubs, and other such permanent and all-comprehending institutions. Above and before all, as being the most comprehensive and least controversial of all, he should know with the most detailed thoroughness everything about the hospital of his Parliamentary district. Tried by these simple tests, how many Members of Parliament would come out in the first class list?

It is the conviction of many who are well qualified to judge that hospitals are to play a much more important and conspicuous part in the community in the future than they have done in the past. Experience is steadily convincing the world of the pre-eminent value of careful and skilled nursing, and of perfect hygienic conditions in the treatment of disease as compared with the mere use of drugs. To leave a patient, suffering from a severe attack of typhoid fever or pneumonia, in such a home and under such circumstances of incompetent nursing and management as vast numbers of artisans and small tradesmen are accustomed to, is to ensure that patient's death. To remove him to a wholesome, well-appointed hospital, where nursing and management are of the best, is in all cases to give him a chance of life, and in many to make recovery a certainty. The same may be said of many other diseases as well as of typhoid fever and pneumonia. The time will probably come when all serious illness among the lower middle-classes will be treated in hospitals.

But if that be so, a revolution awaits the hospital system of the present day. That revolution must not be left to evolve itself by chance and accident. It must be taken in hand by those who possess the knowledge of men, the trained aptitudes of the statesman, the broad outlook of the experienced man of the world. The average hospital manager or physician has little experience of affairs, little opportunity of rising above the level of his surroundings in knowledge and judgment. Not unnaturally he learns to think that he knows more about hospital management than all other people. And so perhaps he does about the domestic and financial details of his own particular institution. But there are now wanted men of statesmanlike capacity who are able to deal, not merely with one hospital, but with the whole system of hospitals, with the whole immense question of the medical management of the sick and incapable poor. A workhouse master or a relieving officer may be the most unfit man in the world to preside at the poor-law board; and similarly a hospital official may be entirely incompetent to take a comprehensive survey and map out a general scheme for the conduct of the whole hospital system.

Members of Parliament are presumably men of some general capacity. They should have eyes to see what Tennyson has called the "main currents which draw the years." If the revolution we have hinted at be accomplishing itself, as we are sure it is, and the bulk of grave diseases among the industrial populations are ultimately to drift to the hospital, serious changes must be apprehended in the affairs of vast numbers of the people. The question is not one which merely affects poor patients. It affects persons who are much higher in the social scale, and who constitute a numerous and important class. What will the medical men who now live by the fees of the lower middle class say to the change? And what will those who support hospitals say? The former will cry out, and do cry out already, that they are being ruined by the competition of the hospitals; the latter complain, and not without good reason, that in the midst of the present bad times the hospitals are placing on their shoulders a heavy burden and grievous to be borne.

The permanent continuance of such a condition of things is impossible. Doctors say they are being ruined; subscribers say they are being exhausted beyond power of recuperation; and patients by their dumb and sorrowful necessities, say that they must not in a Christian country be left to die so long as help is possible. There is therefore a clear, urgent, and large necessity for the immediate intervention of men of judgment, capacity, and courage. This is not an affair of Christmas doles or of village soup kitchens. It is a perennial care which belongs to the whole community, and which, if neglected or unintelligently handled, means permanent loss of health and the power of self-support to many, whilst to not a few it is nothing less than death.

Each Member of Parliament has probably at least one hospital within the limits of his constituency. Some have several. As a preparation for understanding the whole system and the necessities of the poor as regards that system, he should make himself familiar with the actual working of the hospital which may be said to be his own. That would furnish him with a kind of object lesson, a multiplication table, an algebraic formula which would help him by persevering labour to acquire a large and intelligent knowledge of the whole question. Some other countries are decidedly ahead of us in competent and effective management of their sick. It remains for those who claim to be public men to show by clear understanding of what questions are really of public importance, and by intelligent and capable handling of such questions, that this country too is able to cope with the great problems of a complex civilization, and thereby to prove its fitness and power to survive. The doctors must be reconciled to the hospitals, the subscribers must have their burdens readjusted and made possible for their strength, the toilers who form the basis of our social organism, and without whom social wellbeing is impossible, must not have less but more help, and that help must be more efficient and more prompt. These are the difficulties which lie before us; these the problems which press for immediate solution; and we call upon Members of Parliament as well as upon all other clear-sighted men to put their hands to the work without delay.

